# Supplementary material for: Design optimization of high-sensitivity PCF-SPR biosensor using machine learning and explainable AI
Source: PLoS One. 2025 Sep 15;20(9):e0330944. doi: 10.1371/journal.pone.0330944 (PMC12435684; doi:10.1371/journal.pone.0330944)
Supplement: Attachment — Submitted filename: Review-25-28444.docx [file pone.0330944.s001.docx]

**Article: Design optimization of high-sensitivity PCF-SPR biosensor using machine learning and explainable AI**

**Comments to Authors**

**Technical**

1. Explain how the integration of machine learning regression techniques contributes to the optical property prediction in the proposed PCF-SPR biosensor and with the machine leaning approaches can you be precise in finalizing the final sensor parameters.
2. I suggest presents a detail about various AI based techniques that can be used in the sensor optimization. Follow the following article to presents details of various AI based techniques used in sensor optimization and cite the article. *"Finite Element Method-Based Modeling of a Novel Square Photonic Crystal Fiber Surface Plasmon Resonance Sensor with a Au–TiO2 Interface and the Relevance of Artificial Intelligence Techniques in Sensor Optimization"*
3. Discuss the significance of the broad RI detection range (1.31 to 1.42) in the context of biosensing applications, justify the RI range since any RI below 1.33 is considered superficial, I suggest add a sentence like claiming theoretical analysis.
4. How does the use of SHAP (SHapley Additive exPlanations) enhance the interpretability of the ML model used for sensor design optimization?
5. Analyze how the achieved performance metrics such as wavelength sensitivity and figure of merit reflect the sensor's capability for high-precision detection.
6. Are these any sensing parameters which cannot be predicted using ML models, and why, and how do these predictions benefit the overall sensor design process?
7. Critically evaluate the influence of design parameters like Au thickness and pitch on the sensor's performance, as revealed by the SHAP analysis.
8. Compare the advantages of the hybrid design approach combining conventional methods and ML/XAI with traditional sensor optimization strategies in terms of efficiency and computational cost. Some prominent sensor models having merger of IMD and EMD, merger of Quasi D shaped with EMD approach etc need to be included in the comparison section of the article. Add a short paragraph suggesting future application of the sensor for various applications. Following are suggested references including these features.

<https://doi.org/10.1007/s11468-024-02400-7>

https://doi.org/10.1007/s11082-023-05016-z

https://doi.org/10.1016/j.ijleo.2022.169892

https://doi.org/10.1016/j.measurement.2021.110513

9. Justify the suitability of the proposed biosensor for medical diagnostics and chemical sensing, particularly in the context of cancer cell detection, refer the following reference.

https://doi.org/10.3390/bios15050292

**Comments to Editor**

Dear Sir, Good Morning

Sir author have done good work in the article my comments will further enhance the work only. I suggest acceptance of article with "minor revision".

Thanks
