# Supplementary material for: Design optimization of high-sensitivity PCF-SPR biosensor using machine learning and explainable AI
Source: PLoS One. 2025 Sep 15;20(9):e0330944. doi: 10.1371/journal.pone.0330944 (PMC12435684; doi:10.1371/journal.pone.0330944)
Supplement: Attachment — Submitted filename: PONE D-25 Comments.docx [file pone.0330944.s002.docx]

**Article: Design optimization of high-sensitivity PCF-SPR biosensor using machine learning and explainable AI**

**Review for Authors**

1. Explain how machine learning and artificial intelligence-based techniques can be used in the sensor design optimization and design. I suggest go through the following article and refer to various techniques used in the sensor modelling cite them too.

<https://doi.org/10.3390/photonics12060565>

<https://doi.org/10.3390/bios15050292>

2. The SHAP (SHapley Additive exPlanations) technique do not enhance the sensor detection capability, it provides more results to conclude, these models are used to analyze the sensor for design optimization purposes only.
3. In Figure 3 and section 2.2 can you see the in the text you have used D1, D2, D3 but in the diagram d1, d2, and where is d3, thus correct the notation. These create confusion.
4. Provide reference to Eq 1, 2, 4, 7, 9 etc, without proper reference it seems like you have developed the equation.
5. In Fig 12,13, 14 can you increase the font size of the x- and y- labels of the figure.
6. Fig 16 don’t look correct, format it well, format all image well increase the font size of the x-label and y-label for all figures.
7. Mention the applications of the proposed biosensor in real time from medical to environment application.

**Comments to Editor**

Dear Sir, Good Afternoon,

The author has conducted commendable work in this article. There are no obvious errors in the article. My comments are intended solely to enhance the quality of the manuscript further. I recommend the acceptance of the article with minor revisions.
