# Supplementary material for: Design optimization of high-sensitivity PCF-SPR biosensor using machine learning and explainable AI
Source: PLoS One. 2025 Sep 15;20(9):e0330944. doi: 10.1371/journal.pone.0330944 (PMC12435684; doi:10.1371/journal.pone.0330944)
Supplement: Attachment — Submitted filename: Response to Reviewers.docx [file pone.0330944.s004.docx]

**Responses to reviewers:**

The authors would like to thank the reviewers for their precious time and valuable suggestions. We have carefully addressed and updated all the changes in the original manuscript. The following section provides point-by-point responses corresponding to all review comments.

**#Reviewer 1**

1. Explain how the integration of machine learning regression techniques contributes to the optical property prediction in the proposed PCF-SPR biosensor and with the machine leaning approaches can you be precise in finalizing the final sensor parameters.

**Response:** We have revised the manuscript to provide a more detailed explanation of how machine learning (ML) regression techniques contribute to the prediction of optical properties in the proposed PCF-SPR biosensor. A new paragraph has been added in the **Section 2.6 (ML Algorithms).**

2. I suggest presents a detail about various AI based techniques that can be used in the sensor optimization. Follow the following article to presents details of various AI based techniques used in sensor optimization and cite the article. *"Finite Element Method-Based Modeling of a Novel Square Photonic Crystal Fiber Surface Plasmon Resonance Sensor with a Au–TiO2 Interface and the Relevance of Artificial Intelligence Techniques in Sensor Optimization"*

**Response:**

In response, we have added a detailed discussion on various AI-based techniques used in sensor optimization, including Artificial Neural Networks (ANNs), classical machine learning models, ensemble methods, and deep learning approaches. Their applications in real-time prediction, inverse design, and performance enhancement have been highlighted. The recommended article has been thoroughly reviewed and cited appropriately in the revised manuscript see **Section 2.5 (AI Techniques for PCF-SPR Sensor Optimization).**

3. Discuss the significance of the broad RI detection range (1.31 to 1.42) in the context of biosensing applications, justify the RI range since any RI below 1.33 is considered superficial, I suggest add a sentence like claiming theoretical analysis.

**Response:** We have explained the reason for choosing the RI range (1.31–1.42) in **Section 2.1.** The lower part of the range (below 1.33) is included to cover some theoretical cases and very light or low-density samples.

4. How does the use of SHAP (Shapley Additive exPlanations) enhance the interpretability of the ML model used for sensor design optimization?

**Response:** We thank the reviewer for emphasizing the importance of model interpretability. To address this, we have added a clear explanation in **Section 2.8** on how SHAP enhances the transparency of the ML model by identifying the impact of each input feature on the predicted optical properties. This helps to guide sensor design decisions and improves overall optimization.

5. Analyze how the achieved performance metrics such as wavelength sensitivity and figure of merit reflect the sensor's capability for high-precision detection.

**Response:** We have now included a detailed explanation in the revised manuscript in **Section 4 (Discussion)** to highlight how the achieved wavelength sensitivity and figure of merit (FOM) substantiate the high-precision detection capability of the proposed PCF-SPR biosensor. The newly added text elaborates on their implications in practical biosensing applications.

6. Are these any sensing parameters which cannot be predicted using ML models, and why, and how do these predictions benefit the overall sensor design process?

**Response:** We have addressed this point in **Section 4 (Discussion)** by discussing which sensing parameters are well-suited for ML prediction and which still require traditional simulation or experimental validation. This addition helps clarify the role of ML in the overall sensor design process and its practical limitations.

7. Critically evaluate the influence of design parameters like Au thickness and pitch on the sensor's performance, as revealed by the SHAP analysis.

**Response:** We have included a focused discussion in **Section 4 (Discussion),** based on SHAP analysis that critically evaluates the influence of design parameters such as gold thickness and pitch. Their respective impacts on key performance metrics like wavelength sensitivity and confinement loss are now clearly highlighted in the revised manuscript.

8. Compare the advantages of the hybrid design approach combining conventional methods and ML/XAI with traditional sensor optimization strategies in terms of efficiency and computational cost. Some prominent sensor models having merger of IMD and EMD, merger of Quasi D shaped with EMD approach etc need to be included in the comparison section of the article. Add a short paragraph suggesting future application of the sensor for various applications. Following are suggested references including these features.

<https://doi.org/10.1007/s11468-024-02400-7>

<https://doi.org/10.1007/s11082-023-05016-z>

<https://doi.org/10.1016/j.ijleo.2022.169892>

<https://doi.org/10.1016/j.measurement.2021.110513>

**Response:** Thank you for the valuable feedback. In response, we have expanded the comparison in **Section 3.3 (Comparison with Previous Works)** of the manuscript to highlight the advantages of our hybrid design strategy combining FEM-based simulation, machine learning (ML), and explainable AI (XAI) techniques over conventional sensor optimization methods. Additionally, we have included comparisons with prominent sensor architectures integrating IMD-EMD and Quasi D-shaped designs, as referenced. A paragraph outlining future real-time applications has also been added in **Section 4 (Discussion)**.

9. Justify the suitability of the proposed biosensor for medical diagnostics and chemical sensing, particularly in the context of cancer cell detection, refer the following reference. <https://doi.org/10.3390/bios15050292>

**Response:** We have added this in **Section 4 (Discussion),** to further elaborate on the medical and chemical sensing relevance of the proposed PCF-SPR biosensor, with a focus on its applicability in cancer diagnostics. The suggested reference has been reviewed and appropriately cited to support our explanation.

**#Reviewer 2**

1. Explain how machine learning and artificial intelligence-based techniques can be used in the sensor design optimization and design. I suggest go through the following article and refer to various techniques used in the sensor modelling cite them too.

<https://doi.org/10.3390/photonics12060565>

<https://doi.org/10.3390/bios15050292>

**Response:** Thank you for the valuable suggestion. We have revised the manuscript to include a detailed overview of how ML and AI techniques contribute to PCF-SPR sensor optimization. This has been added under **Section 2.5 (AI Techniques for PCF-SPR Sensor Optimization)**, along with relevant insights and citations from the suggested references.

2. The SHAP (SHapley Additive exPlanations) technique do not enhance the sensor detection capability, it provides more results to conclude, these models are used to analyze the sensor for design optimization purposes only.

**Response:** We agree with the reviewer’s observation. The manuscript has been updated in **Section 2.8 (Explainable AI (XAI))** to clarify that SHAP does not directly improve sensing performance but helps interpret ML outputs, identify key design parameters, and support informed sensor optimization decisions.

3. In Figure 3 and section 2.2 can you see the in the text you have used D1, D2, D3 but in the diagram d1, d2, and where is d3, thus correct the notation. These create confusion.

**Response:** Thank you for pointing out the inconsistency. The notations in **Figure 3** and **Section 2.2** have been corrected for consistency. All diameter labels now uniformly use the same format (D1, D2, D3), and the missing label (D3) has been added to the figure to avoid confusion.

4. Provide reference to Eq 1, 2, 4, 7, 9 etc, without proper reference it seems like you have developed the equation.

**Response:** References for Equations 1, 2, 4, 7, 9, and others have been added in the revised manuscript to clearly indicate their original sources and avoid any confusion regarding authorship.

5. In Fig 12, 13, 14 can you increase the font size of the x- and y- labels of the figure.

**Response:** Thank you for the suggestion. The font size of the x- and y-axis labels in **Figures 12, 13, and 14** has been increased to improve readability.

6. Fig 16 don’t look correct, format it well, format all image well increase the font size of the x-label and y-label for all figures.

**Response:** Thank you for pointing this out. **Figure 16** has been reformatted for better clarity, and the font sizes of the x- and y-axis labels have been increased. Additionally, all other figures in the manuscript have been reviewed and formatted accordingly to ensure visual consistency and improved readability.

7. Mention the applications of the proposed biosensor in real time from medical to environment application.

**Response:** Thank you for your valuable suggestion. Real-time applications in medical diagnostics and environmental monitoring have been added in **Section 4 (Discussion)** to emphasize the sensor’s practical relevance.
